# Winter severity shapes zooplankton community in a shallow green lake

**DOI:** 10.1002/ecy.70249

**Published:** 2025-12-08

**Authors:** Alia Benedict, Casey Schoenebeck, Thomas Hrabik, Ted Ozersky

**Affiliations:** ^1^ Large Lakes Observatory, University of Minnesota Duluth Duluth Minnesota USA; ^2^ Minnesota Department of Natural Resources Glenwood Minnesota USA; ^3^ Swenson College of Science and Engineering, University of Minnesota Duluth Duluth Minnesota USA

**Keywords:** eutrophication, limnology, oxygen, winterkill, zooplankton

Winter is a biologically active period in seasonally freezing lakes (Hampton et al. 2017). Plankton and fish play important roles in winter processes like nutrient cycling and energy flow which in turn shape open‐water ecosystem dynamics (Sommer et al. 2012). Winter conditions such as lake snow and ice cover can shape winter biological activity and thus the biological connections between seasons (Ozersky et al. 2021; Grosbois et al. 2018).

Winters in the Northern Hemisphere are growing more variable (Sharma et al. 2019), with greater fluctuations in temperature and precipitation within and between winters (Casson et al. 2019; Cohen et al. 2020). Increased intraseasonal variability, such as more frequent rain‐on‐snow events and mid‐winter thaws, can alter light, nutrient, and thermal conditions under lake ice (Kirillin et al. 2012; Engle and Melack 2001), with short‐term impacts on primary production and food web dynamics (Hrycik et al. 2021). Increased interannual winter variability may have more lasting consequences on lake ecosystem function (Feiner et al. 2022). For example, during mild winters, thin snowpack and increased light penetration can advance the spring phytoplankton bloom (Hrycik et al. 2022) with bottom‐up effects on zooplankton and fish (Feiner et al. 2022). Conversely, during severe winters, thick snow and ice cover can limit under‐ice production, promote hypoxia (dissolved oxygen <2 mg/L), and cause mass mortality of fish (Hurst 2007), with bottom‐up and top‐down effects on plankton communities (Schoenebeck et al. 2012; Balayla et al. 2010).

Portage Lake (Hubbard County, MN, USA; Appendix S1: Table S1) is a shallow (mean depth 2.3 m), productive (mean total phosphorus 60 μg/L) lake that generally experiences short, cool summers and long, cold winters. The lake has been sampled annually during the open‐water period since 1987 as part of the Minnesota Sentinel Lakes Program (MPCA 2009) and the Portage Lake Association has tracked ice cover trends for 50 years as well as occurrences of winter fishkills, which are not uncommon in this lake (Appendix S1: Section S1).

As part of a year‐round food web research project, we studied the seasonal development of temperature, oxygen, chlorophyll *a*, and crustacean zooplankton abundance and diversity in Portage Lake during the winters and summers of 2022–2023 and 2023–2024. During both years, sampling was conducted monthly from January to March and again in May, July, and August in the middle of the lake (4.5 m depth) to capture the seasonal development of abiotic and biotic conditions. When present, ice thickness, ice quality, and snow depth were recorded, and complete water‐column profiles of temperature, oxygen, and chlorophyll *a* were taken with a YSI EXO2 multiparameter sonde (YSI Inc., Yellow Springs, OH, USA). Water samples were collected 1 m below the ice‐water interface (or 1 m below the water surface when no ice was present) and 1 m above the lake bottom for discrete chlorophyll *a* analysis. Chlorophyll *a* was filtered onto 0.2‐μm nitrocellulose filters in triplicate, extracted in 90% acetone for 18 h in the dark, and analyzed with a Turner Designs 10‐AU fluorometer. Zooplankton were sampled from 1 m above the lake bottom to the surface using a 25‐cm mouth diameter, 64‐μm mesh net in triplicate, where each replicate consisted of three pooled net tows. Samples were preserved, identified to the lowest identifiable taxon, sorted into major groups, and counted with a stereoscopic microscope under 7×–70× magnification.

The winter of 2022–2023 was particularly severe, with thick snowpack and ice cover that lasted a near‐record period of 5 months and 16 days (average ice duration 5 months; MN DNR 2024). These conditions resulted in a partial winter fishkill as well as an unexpected lake‐wide disappearance of crustacean zooplankton, which did not recover until several weeks after ice‐out. This surprising “zooplankton winterkill” prompted us to return to Portage Lake for further study the following winter. In contrast to the winter of 2022–2023, the winter of 2023–2024 was the region's warmest winter on record, with minimal snowpack and a record short ice cover period of 3 months and 18 days (MN DNR 2024). In just two years of winter study on Portage Lake, we observed stark differences in lake snow and ice cover that coincided with physical, chemical, and biological differences below the ice, which in turn shaped open‐water zooplankton dynamics.

During the severe winter of 2022–2023, Portage Lake was covered by white, opaque ice and thick snow (Appendix S1: Figure S1) which coincided with low water temperatures (Appendix S1: Figure S2), low chlorophyll *a* concentrations, and a seasonal decrease in dissolved oxygen (Figure 1). Forty‐seven days after ice‐on (3 January 2023), dissolved oxygen was available throughout the water column, and chlorophyll *a* concentrations were high under the ice. Zooplankton were abundant at 24 individuals per liter (Ind/L), diverse, and consisted of small cladocerans, large and small adult copepods, and copepod juveniles (Figure 2a; Appendix S1: Table S2). Eighty‐eight days after ice‐on (13 February), dissolved oxygen and chlorophyll *a* concentrations declined significantly and water collected from the lake center at 4.5 m smelled strongly of hydrogen sulfide, a compound toxic to invertebrates and fish (Dunnette et al. 1985). Total zooplankton abundance decreased to 17 Ind/L and consisted only of adult and juvenile copepods. One hundred twenty‐eight days after ice‐on (25 March), dissolved oxygen and chlorophyll remained low. At this point, zooplankton abundance declined to 0 Ind/L and we observed only occasional copepod juveniles and small cladocerans in the pelagic and littoral zones. This was based on three routine samples collected at lake center, as well as five additional investigative samples taken throughout the lake. The collapse of zooplankton coincided with observations of dead fish, and dead or sluggish benthic invertebrates in pelagic and littoral sediments. Following ice‐out on 3 May, the zooplankton community had recovered to 8 Ind/L and was composed primarily of small copepod juveniles. Peak abundance occurred in late May at 202 Ind/L, where the community was dominated by small copepod juveniles. Abundance then declined throughout the open‐water period, and the community shifted toward larger zooplankton before returning to predominantly small copepod juveniles in early August (Figure 2a).

**FIGURE 1:**
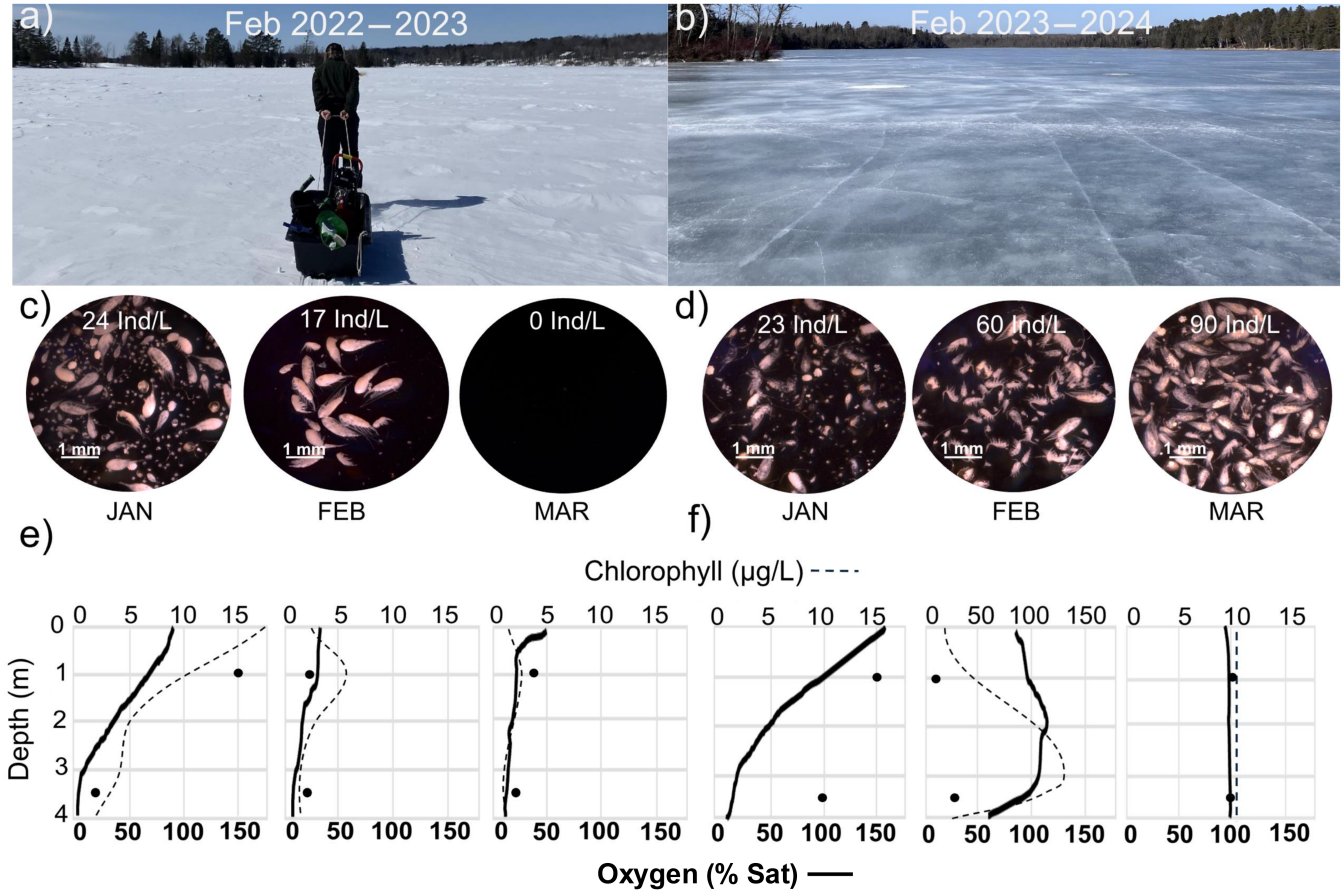
Physical, chemical, and biological parameters in Portage Lake during a severe and a mild winter. (a, b) Physical conditions on Portage Lake during the winters of 2022–2023 and 2023–2024. (c, d) One milliliter aliquots of homogenized zooplankton samples collected from the pelagic zone during January, February, and March in 2022–2023 and 2023–2024. Abundance in individuals per liter (Ind/L) is averaged from three replicates per sample point. 1 mm =1 millimeter. (e, f) Seasonal trends of dissolved oxygen in percent saturation (% Sat, bold line) and chlorophyll *a* in micrograms per liter (μg/L, dashed line) from singular sonde casts in the pelagic zone in 2022–2023 and 2023–2024. Discrete chlorophyll *a* values (μg/L) are indicated by closed circles. No chlorophyll‐*a* CTD data in January 2024. Note the scale difference for chlorophyll *a* in February 2024. Photo credits: Alia Benedict.

**FIGURE 2:**
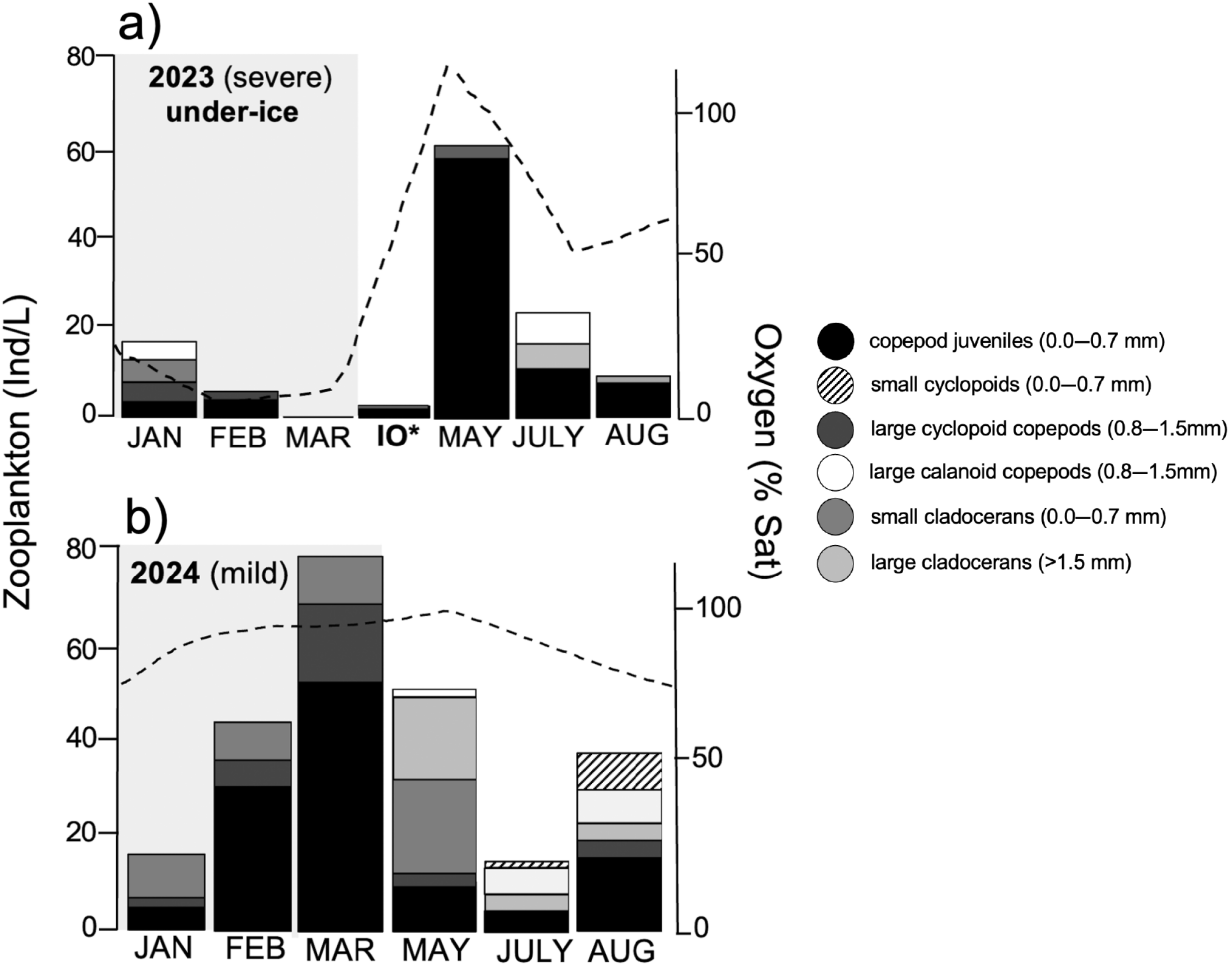
Seasonal changes in crustacean zooplankton abundance and diversity and averaged full‐water column measurements of dissolved oxygen in percent saturation (% Sat, dashed line) in the pelagic zone of Portage Lake during a severe and a mild winter. Abundance values in individuals per liter (Ind/L) are averaged from three replicates per sample point. IO indicates ice‐out on 3 May 2023. Zooplankton size classes determined from Balcer et al. 1984. mm = millimeter.

During the mild winter of 2023–2024, Portage Lake was covered by thin, translucent ice with minimal snow cover (Appendix S1: Figure S1), which coincided with elevated water temperatures (Appendix S1: Figure S2), high chlorophyll a concentrations, and high dissolved oxygen throughout the winter, as well as an abundant and active zooplankton community under the ice (Figure 1). Forty‐eight days after ice‐on (14 January 2024), dissolved oxygen was supersaturated under the ice and chlorophyll concentrations were high throughout the water column. Zooplankton were abundant at 23 Ind/L and the community was dominated by small cladocerans, large, egg‐carrying copepods, and juvenile copepods (Figure 2b; Appendix S1: Table S2). Eighty‐nine days after ice‐on (24 February), oxygen and chlorophyll *a* increased throughout the water column, and zooplankton abundance increased to 60 Ind/L. One hundred twenty‐one days after ice‐on (28 March), partial ice‐out, lake mixing, and re‐freezing occurred, which produced areas of new, thin ice along the shores. Dissolved oxygen and chlorophyll *a* remained high throughout the water column and we observed the greatest zooplankton densities of any sample point that year at 90 Ind/L. Complete ice‐out occurred on 6 April 2024. Zooplankton abundance declined over the summer, before increasing again in late August, where the summer community was composed of a diverse mix of large and small adult cladocerans and copepods (Figure 2b).

What caused the disappearance of crustacean zooplankton during the severe winter of 2022–2023? Our observations strongly suggest that the hypoxic conditions that developed throughout the winter caused widespread zooplankton mortality. Studies on the hypoxia tolerance of common zooplankton taxa show the sensitivity of many groups, including large‐bodied cladocerans and copepods, to low oxygen concentrations (Vanderploeg et al. 2009; Karpowicz et al. 2020), which aligns with our observation that large‐bodied zooplankton were less abundant during and after the severe winter and more abundant during and after the mild winter. The negative effect of hypoxia on zooplankton is further supported by the fact that only hypoxia‐tolerant groups, such as copepod juveniles and small cladocerans (Stalder and Marcus 1997; Karpowicz et al. 2020) were observed in late winter samples in 2022–2023.

An alternative explanation for the zooplankton collapse may be the reduced phytoplankton biomass during the severe winter of 2022–2023. While winter phytoplankton are important for zooplankton survival (Grosbois et al. 2017), it is not likely that their reduced biomass caused the observed disappearance of crustacean zooplankton. Chlorophyll *a* concentrations during the severe winter were comparable to those in other lakes with robust winter zooplankton populations (Hampton et al. 2017; Shchapov et al. 2021) and higher than those from a nearby oligo‐mesotrophic lake that did not experience winterkill during the same period (Pike Lake, St. Louis County, MN, USA; Benedict, unpublished data).

Severe winters can cause hypoxia and fishkills in shallow, green lakes (Greenbank 1945; Hurst 2007; Balayla et al. 2010), which can ease top‐down control on lake food webs by fish predators (Ruuhijärvi et al. 2010; Balayla et al. 2010). Direct observations of zooplankton responses to winter hypoxia, however, are rare. Greenbank (1945) noted a ‘practical extinction’ of zooplankton in the Illinois River following a winter fishkill, and Isermann et al. (2004) and Schoenebeck et al. (2012) found low zooplankton abundance in shallow winterkill lakes following severe winters. While winter fish mortality is a known top‐down control on lake food webs, a partial or complete reduction of winter zooplankton, which is likely more common than currently described, may have alternative, bottom‐up effects on nutrient cycling and food web dynamics.

Here we directly document the response of a lake zooplankton community to two different winters, showing the strong and likely impact of low winter oxygen concentrations on zooplankton abundance, diversity, and the timing of the seasonal zooplankton peak. During the severe winter, zooplankton were less abundant, less diverse, and smaller, with peak abundance occurring after ice‐out, which preceded an open‐water community dominated by small copepod juveniles. During the mild winter, zooplankton were more abundant, more diverse, and larger, with peak abundance occurring under the ice, which preceded a diverse summer community of multiple groups and size classes. Although low oxygen concentrations during the severe winter are likely the primary driver of the observed differences in the winter zooplankton community, elevated summer water temperatures following the mild winter (Benedict 2025) may have also influenced the patterns observed in the summer community. In both cases, our results indicate that winter severity, and importantly, interannual winter variability, play a large role in shaping the ecosystems of shallow, green lakes.

Our observation of a likely zooplankton winterkill raises several key questions. How often do such winterkills occur, and to what extent do they influence the structure and dynamics of freshwater communities? What impact do low winter oxygen concentrations have on other overwintering communities, such as phytoplankton or benthos? How widespread are these events across lakes that differ in size, depth, and trophic status, and what are their long‐term impacts on lake food webs and ecosystems? Emerging research on the consequences of warming winters indicates that changes in ice cover duration and quality influence oxygen dynamics in ways that vary across regions and lake types (Jansen et al. 2025; Ozersky et al. 2025). Direct observations of ice cover, ice quality, and under‐ice biological activity across diverse lake types are needed to better understand how changing winters will affect lake ecosystems.

In addition to documenting an interesting natural history phenomenon, our observations highlight the dynamic nature of winter lake ecosystems and the importance of winter conditions to whole‐year ecosystem function. We show that winter lake habitats are not static during the ice cover period, but rather change throughout winter and between years. We sampled Portage Lake during two consecutive and very different winters, and we observed large intra‐ and interannual differences in temperature, oxygen, and chlorophyll *a* that significantly influenced the winter zooplankton community. Recent work in winter limnology has emphasized that life is active under lake ice. The next steps are to understand how winter variability influences physical, chemical, and biological processes beneath the ice, and how these dynamics shape conditions during the open‐water season.

## CONFLICT OF INTEREST STATEMENT

The authors declare no conflicts of interest.

## Supporting information


Appendix S1.

## Data Availability

Data (Benedict et al. 2025) is available in Dryad at https://doi.org/10.5061/dryad.m63xsj4fs.
