# Supplementary material for: Winter severity shapes zooplankton community in a shallow green lake
Source: Ecology. 2025 Dec 8;106(12):e70249. doi: 10.1002/ecy.70249 (PMC12683618; doi:10.1002/ecy.70249)

Appendix S1

Winter severity shapes zooplankton community in a shallow green lake

Alia Benedict, Casey Schoenebeck, Thomas Hrabik, and Ted Ozersky

Ecology

Section S1

Portage Lake Association. *Portage Lake Ice Dates, Hubbard County*. https://www.peteport.com/portage/ice%20dates.html

Portage Lake Association. *Fall 2014 Portage Lake Newsletter*. https://www.peteport.com/portage/2014FallPortageNewsletter.pdf

Table S1. Location and characteristics of Portage Lake (Hubbard County, MN, USA). Water quality parameters are total mean values from the open-water period.

Lake, County	Elevation (m.a.s.l.)	Area (acres)	Percent Littoral	Max Depth (m)	Mean Depth (m)	Secchi Depth (m)	TP (µg/L)	Chl. *a* (µg/L)
Portage, Hubbard	437	422	96%	5.2	2.3	3	60	21

Table S2. Prominent zooplankton taxa present in Portage Lake during winters 2022-2023 and 2023-2024 and the following open-water periods. Only taxa with ~10% or greater occurrence in samples noted. Copepodites and nauplii are noted separately but considered jointly as ‘copepod juveniles’ in the text.

Portage Lake	2022-2023	2023-2024
January	*Bosmina longirostris, Chydorus sphaericus, Diacyclops thomasi, Tropocyclops prasinus mexicanus,* copepodites, nauplii	*B. longirostris, D. thomasi,* copepodites, nauplii
February	*D. thomasi,* copepodites, nauplii	*B. longirostris, D. thomasi,* copepodites, nauplii
March	Copepodites present at 0.1 Ind/L	*B. longirostris, D. thomasi,* copepodites, nauplii
Ice-off	*D. thomasi,* copepodites, nauplii	No data
May	Copepodites, nauplii	*D. mendotae, C. sphaericus,* copepodites, nauplii
July	*Daphnia mendotae, Skistodiaptomus oregonensis,* copepodites	*D. mendotae, S. oregonensis, T.p mexicanus,* copepodites, nauplii
August	Copepodites, nauplii	*T.p. mexicanus,* copepodites, nauplii

Figure S1. Monthly development of snow and ice cover on Portage Lake during a severe and mild winter. Photo credits: Alia Benedict.

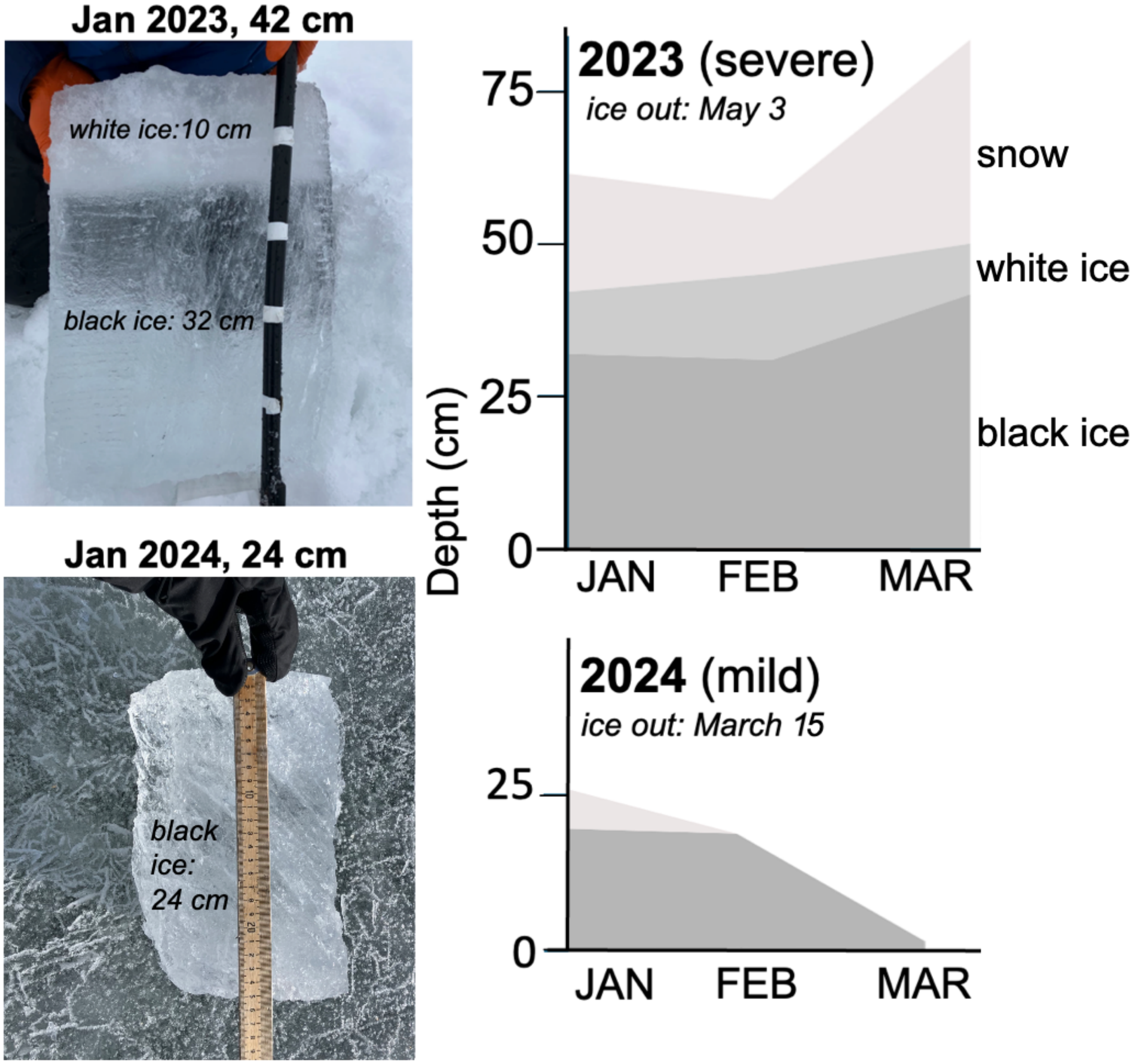

Figure S2. Monthly temperature profiles in the pelagic zone of Portage Lake during a severe and mild winter. Lake mixing occurred shortly before the March 2024 sample event.

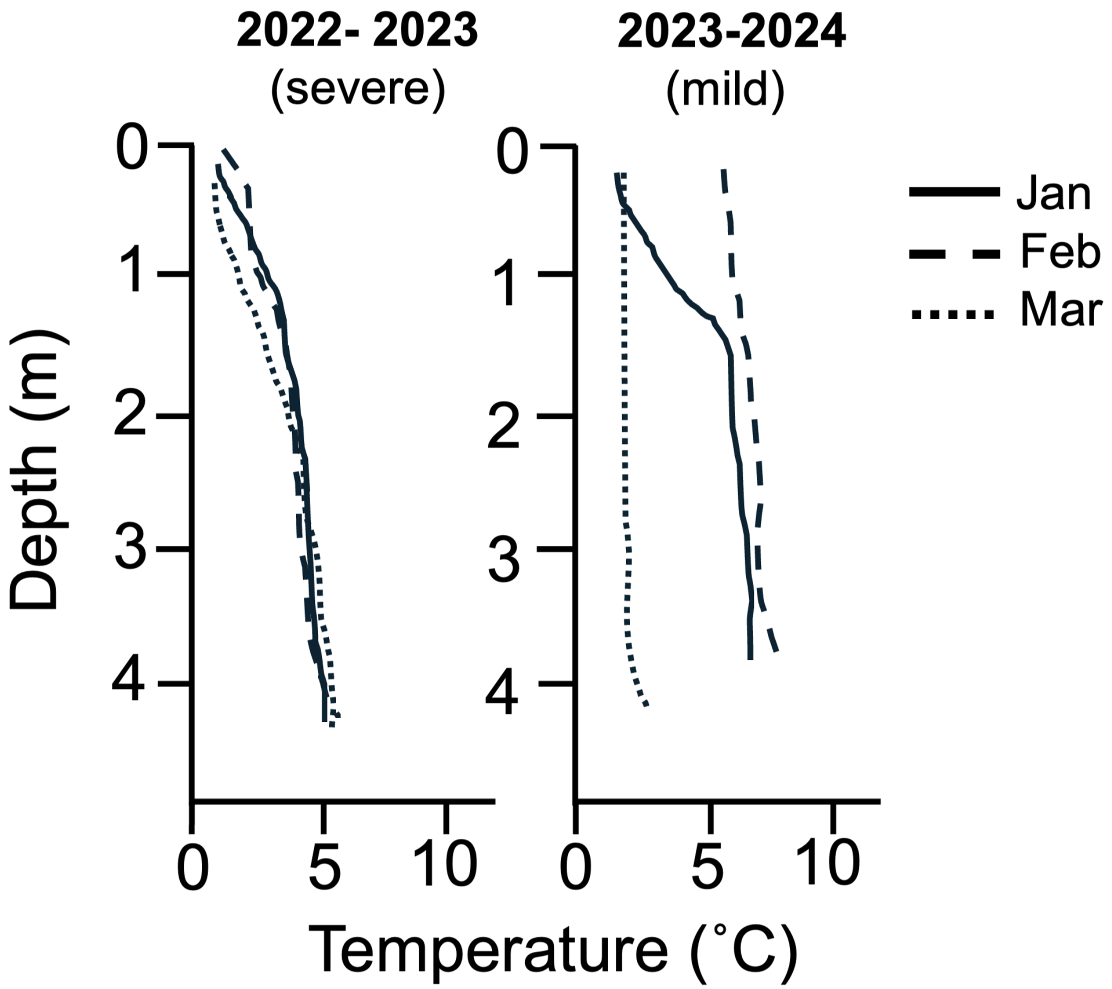

Supplement: Supplementary file 1 — Appendix S1. [file ECY-106-e70249-s001.pdf]
